# Let’s talk about recovery in mental health: an international Delphi study of experts by experience

**DOI:** 10.1017/S2045796024000490

**Published:** 2024-09-24

**Authors:** E. Guerrero, M. Barrios, H. M. Sampietro, A. Aza, J. Gómez-Benito, G. Guilera

**Affiliations:** 1 Department of Social Psychology and Quantitative Psychology, Institute of Neurosciences, University of Barcelona, Spain; 2 Department of Social Psychology and Quantitative Psychology, University of Barcelona, Spain; 3 ActivaMent Catalunya Associació, Barcelona, Spain; 4 Department of Personality, Evaluation and Psychological Treatments, University of Salamanca, Spain

**Keywords:** mental health, models/theories of psychiatry, psychiatric services, rights of persons with disabilities, social and political issues

## Abstract

**Aims:**

The concept of recovery is featured in the strategic plans of the World Health Organization as well as in other national mental health plans; however, there have been differing interpretations of what it means. This article aims to achieve a consensus on the key aspects of recovery in mental health from the perspective of movements of users and survivors of psychiatry at an international level. Four specific objectives were proposed in this study: (1) to identify what recovery in mental health means, (2) to identify the indicators that a person is progressing in their recovery, (3) to determine the factors that facilitate the recovery process, and (4) to determine the factors that hinder the recovery process.

**Methods:**

A three-round e-Delphi study was conducted with the participation of 101 users and survivors of psychiatry, adhering to the CREDES checklist to ensure methodological rigour.

**Results:**

The results reveal 26 key aspects that define recovery, 31 indicating that a person is progressing in their recovery process, 8 that facilitate recovery and 12 that hinder recovery. The most agreed-upon statements for defining recovery highlight the importance of empowerment, leading a fulfilling life, ensuring safe-living conditions and acknowledging individuals as holders of rights. Similarly, empowerment and agency were highly agreed upon as relevant recovery indicators. Key findings underscore the significance of a supportive and respectful social environment in facilitating recovery, while coercion, discrimination and lack of support from significant others hinder recovery.

**Conclusions:**

Despite cultural differences and recovery’s subjective nature, our results demonstrate that an international consensus on critical recovery aspects is attainable. Highlighting a significant shift, we emphasize the ‘Transition’ process to signify moving away from the biomedical model approach and advocating for collective rights. Our findings advocate for empowerment, users’ rights and the move towards person-centred care that integrates social, political and economic contexts. These consensus statements lay the groundwork for future research across diverse regions and cultures, offering insights into recovery’s meaning and potential for innovative approaches in diagnosis, intervention and evaluation.

## Introduction

The recovery concept is included in the strategic plans of the WHO and the mental health plans of numerous countries; nonetheless, various interpretations of its meaning have been reported (Shepherd *et al.*, 2008). Over two decades, literature on mental health recovery highlights at least two different interpretations: clinical and personal recovery (Leamy *et al.*, 2011; Slade *et al.*, 2012; van Weeghel *et al.*, 2019). Clinical recovery, emerging from professional-led research and practice (Schrank and Slade, 2007; Slade *et al.*, 2012), emphasizes symptom absence and pre-illness functioning (Piat *et al.*, 2009; Schrank and Slade, 2007). Personal recovery, developed in the context of deinstitutionalization and civil rights movements of users and survivors of psychiatry, advocates for self-determination and opposes involuntary admissions and forced treatment (Schrank and Slade, 2007). From this latter perspective, the elimination or reduction of symptoms and the return to previous or ‘normal’ levels of functioning are no longer the principal aims of interventions (Schrank and Slade, 2007; van Weeghel *et al.*, 2019).

To elucidate the meaning of recovery, in 2011, Leamy and colleagues conducted a systematic review of 97 publications defining the concept of personal recovery, resulting in the widely recognized CHIME framework (Kuek *et al.*, 2020), representing (a) Connection with others and with the community; (b) Hope and optimism about the future; (c) a positive sense of one’s own Identity; (d) Meaning and purpose in life and (e) Empowerment. Recently, the SPICE model (Vera San Juan *et al.*, 2021) proposed recovery as a four-dimensional concept that includes Social recovery, Prosperity, Individual recovery and Clinical recovery experience.

In addition to advances in theoretical models used to conceptualize recovery, other studies have been interested in directly asking users of mental health about the meaning of recovery from their perspective (Kidd *et al.*, 2014; Law and Morrison, 2014; Piat *et al.*, 2009). Despite the subjective nature of the personal meaning of recovery, it seems to have common themes behind users’ experiences (Slade *et al.*, 2014).

While the personal recovery-oriented care approach originated from the movements of users and survivors of psychiatry and their critiques of the biomedical model, there remains a lack of consensus in defining recovery from these movements’ perspectives. Therefore, this study aimed to achieve a consensus on the key aspects of recovery in mental health from the perspective of movements of users and survivors of psychiatry at an international level. Four specific objectives were proposed in this study: (1) to identify what recovery in mental health means, (2) to identify the indicators that a person is progressing in their recovery, (3) to determine the factors that facilitate the recovery process and (4) to determine the factors that hinder the recovery process.

## Methods

### Study design

To achieve consensus on the key aspects of recovery in mental health among users and survivors of psychiatry, this study utilized the e-Delphi method (Donohoe *et al.*, 2012). Following the methodology employed in similar studies within the mental health context (Koekkoek *et al.*, 2009; Langlands *et al.*, 2008; Law and Morrison, 2014), our Delphi study was structured in three rounds, hosted on Qualtrics XM Software platform. It began with an initial round featuring open-ended questions to gather a wide array of viewpoints from participants, followed by two additional rounds aimed at achieving consensus. The second and third rounds presented questionnaires with a Likert-type scale to rate the relevance of statements derived from the responses to the open-ended questions of the first round.

The methodological rigour was maintained by consistently following the CREDES(i.e., Conducting and Reporting Delphi Studies) checklist during the study (Jünger *et al.*, 2017).

### Participants

The participant inclusion criteria were as follows: (a) being over 18 years of age; (b) identifying oneself as a user of mental health services or as a survivor of psychiatry and (c) either being a member of an organization of users of mental health services and survivors of psychiatry or having authored technical or scientific publications from a position of an expert by lived experience.

To create a list of potential users and survivors, four recruitment strategies were considered, including the identification of (1) organizations of users and survivors of psychiatry and affiliated members, (2) authors of technical or scientific publications, (3) contributors to the QualityRights materials and (4) additional participants through snowball sampling. For detailed information about the process followed, see the online Supplementary Table S1. As a result of strategies (1), (2) and (3), 53 organizations of users and survivors, and 128 users and survivors of psychiatry were invited to participate in the study. As for strategy (4), these organizations were required to share information about this study with their affiliates and advertise the study to other organizations worldwide. Similarly, users and survivors who consented to participate in this study were asked to share the contact information of others who might be interested in participating.

The study’s invitation email was distributed in English and Spanish, while the informed consent and sociodemographic questionnaire were available in English, French, Russian and Spanish, reflecting the linguistic diversity of the initial contact list. Participants could also request materials in additional languages as needed. According to their language preferences, the Delphi rounds materials were provided in English, Mandarin and Spanish.

### Data collection

Data were collected from September to November 2022. Participants were emailed the questionnaire link and instructions, with invitations for each round followed by two reminders every 3 days. Based on feedback, a 3-day extension was granted per round for non-respondents. After the extended deadline, the questionnaire closed, preventing further responses. Each round lasted approximately 2 weeks.

The first round consisted of four open-ended questions about their experience in recovery: what does recovery in mental health mean to you? (Question 1), what tells you that a person is making progress in their recovery? (Question 2), what factors facilitate the recovery process? (Question 3) and what factors hinder the recovery process? (Question 4). These four questions were developed to pursue the four objectives of the present study, based on insights from van Weeghel *et al.*’s (2019) systematic review and statements from a prior Delphi study (Law and Morrison, 2014), in collaboration with the research team, including a peer researcher with lived experience. All responses were coded through inductive coding to create ad hoc categories from the data without prearranged topics.

In the second round, statements derived from coding responses to the initial four questions were presented separately. Participants were required to rate the relevance of each statement using a Likert-type scale (i.e., ‘not relevant’, ‘slightly relevant’, ‘moderately relevant’, ‘relevant’ and ‘very relevant’).

The third round included statements that 70%–79% of participants rated as ‘relevant’ and ‘very relevant’ in the second round. Once again, the questionnaire was structured based on the first-round questions, and participants re-evaluated the statements using the same Likert-type scale. They were also shown their previous ratings and the percentage of participants that rated each statement as ‘relevant’ and ‘very relevant’ in the second round (see the online Supplementary Figure S1). Only participants who answered the second round were invited to complete the third questionnaire.

### Data analysis

Since participants could respond in any language, first-round responses in languages other than English or Spanish (e.g., one participant responded in Mandarin) were translated into English using an automated translation service and reviewed by one researcher and one proficient collaborator (see the Acknowledgements section). Responses were uploaded to ATLAS.ti Web (version 22.2.4-2022-09-28) for organization and categorization. We conducted a qualitative analysis through inductive coding, following Braun and Clarke’s thematic analysis approach (Braun and Clarke, 2006; Clarke and Braun, 2016). This method generated ad hoc categories directly from the data, without relying on prearranged topics. Following Patton’s (1999) recommendations for consistency, we used an analyst triangulation procedure: two researchers independently coded the responses, which were then compared and reconciled with a third coder to resolve discrepancies. This resulted in a comprehensive list of categories later converted into statements to enhance clarity (see the online Supplementary Table S2). Finally, the statements were presented to the entire research team to review them, remove redundancies and improve writing.

After the second round, we calculated the percentage of agreement regarding the relevance of each statement following the recommended criteria (Barrios *et al.*, 2021; Langlands *et al.*, 2008; Law and Morrison, 2014). Statements rated as ‘relevant’ or ‘very relevant’ by 80% or more of participants passed the cut-off point and were considered as agreed upon by consensus. Statements rated as ‘relevant’ or ‘very relevant’ by 70%–79% of participants were selected to be re-rated in the third round. Statements not meeting these conditions were discarded (see the online Supplementary Table S3).

Similarly, after the third round, we calculated the percentage of agreement regarding the relevance of each statement. Statements rated as ‘relevant’ or ‘very relevant’ by at least 80% of participants were considered as agreed upon by consensus, while those not reaching this cut-off were discarded.

## Results

**Figure 1.**
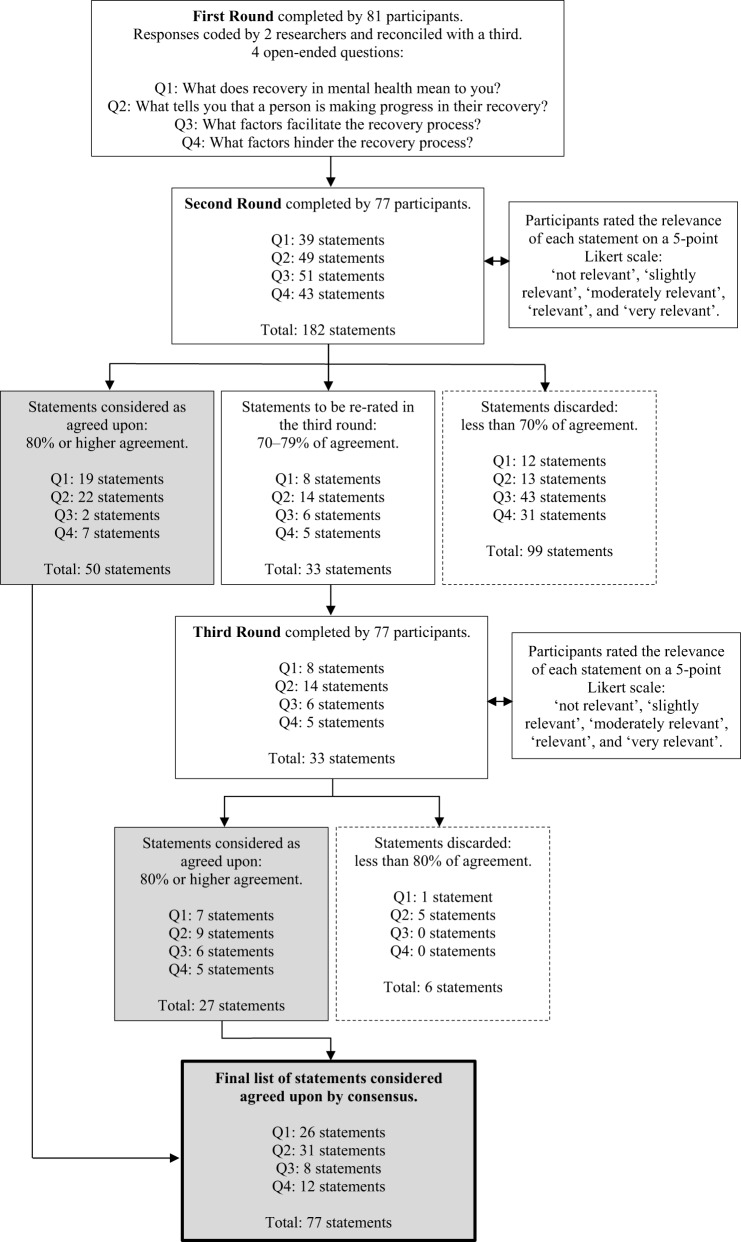
The Delphi process.

Following the initial recruitment (53 organizations and 128 users and survivors), 11 organizations of users and survivors (20.75%) confirmed their collaboration by sharing the invitation with their members, and 34 users and survivors of psychiatry (26.56%) confirmed their participation. Ultimately, a total of 101 users and survivors of psychiatry agreed to participate in the study. Summary statistics for the sociodemographic characteristics of participants (*n* = 101) are displayed in Table 1. Seventy-seven participants completed the three rounds of the study. The results show 77 statements agreed upon by consensus over three rounds of questioning in this Delphi study. Figure 1summarizes the results obtained in each of the three rounds.

**Table 1.** Distribution of sociodemographic characteristics of participants

Variables	Participants (*N* = 101)
Age (years) *M* (*SD*)	47.8 (10.8)
Gender *n* (%)	
Female	52 (51.5)
Male	44 (43.6)
Non-binary	1 (1.0)
Not reported	4 (4.0)
Continents of origin *n* (%)	
Africa	6 (5.9)
America	
Central and South America	2 (2.0)
North America	9 (8.9)
Asia	7 (6.9)
Europe	
Eastern Europe	2 (2.0)
Northern Europe	9 (8.9)
Southern Europe	57 (56.4)
Western Europe	4 (4.0)
Oceania	5 (5.0)
Organization involvement *n* (%)	
Yes	81 (80.2)
No	20 (20.8)

Abbreviations: M, mean; SD, standard deviation.

Table 2 presents the 26 statements used to define recovery (i.e., Question 1), with their percentage of agreement according to the relevance of each statement and the round number in which agreement was obtained. The statements with the highest percentage of agreement regarding their relevance point to developing empowerment, developing a fulfilling life, having safe living conditions and being recognized as a subject with rights.

**Table 2.** Definition of recovery: statements with more than 80% of agreement

Statement^a^	Agreement (%)	Round
1. Developing empowerment (i.e., regaining control of one’s own life).	94.8	2
2. Developing a fulfilling life.	94.8	2
3. Feeling safe (e.g., having conditions needed to live).	94.8	2
4. Being recognized as a subject with rights (e.g., one’s limits and decisions are respected).	94.8	2
5. Having self-determination (i.e., being the protagonist of one’s process, making decisions about one’s own identity and future, without others’ control)	93.5	2
6. Feeling good about oneself (e.g., having a positive identity, self-esteem).	92.2	2
7. Overcoming self-stigma (e.g., sick role).	91.3	3
8. Improving wellness (e.g., reducing distress).	90.9	2
9. Rebuilding life (e.g., relationships, employment) after a mental health crisis.	90.9	2
10. Having self-awareness (i.e., assuming one’s capacities and difficulties).	89.9	3
11. Defending collective rights (e.g., against psychiatric violence or social barriers).	89.9	3
12. Developing a sense of agency (i.e., making meaningful decisions in life).	89.6	2
13. Having support from significant others (e.g., family, friends).	88.3	2
14. Growing as an individual on different levels (i.e., thriving).	87.0	2
15. Having hope (i.e., being hopeful about the future).	87.0	2
16. Getting a personal meaningful purpose out of one’s own experience.	85.7	2
17. Achieving the desired quality of life (e.g., having well-being).	85.7	2
18. Having a life goal.	85.5	3
19. Enjoying leisure and free time.	84.4	2
20. Moving away from the dominant biomedical model approach (i.e., emphasis on diagnosis, clinical input, involuntary detention and forced treatment).	84.1	3
21. Coping with daily life demands.	83.1	2
22. Having a satisfactory social life (e.g., being accepted by others, enjoying relationships).	83.1	2
23. Having personal autonomy (i.e., independent living).	83.1	2
24. Developing resilience.	82.6	3
25. Having a personal path.	81.2	3
26. Dealing with mental health problems (e.g., asking for help, preventing relapse).	80.5	2

a Statements rated as ‘relevant’ or ‘very relevant’ by more than 80% of the participants after the second and third rounds.

Corresponding to the indicators that a person is progressing in their recovery (i.e., Question 2), 31 statements were rated as ‘relevant’ or ‘very relevant’ by at least 80% of participants. Table 3 displays these statements ordered by the percentage of agreement obtained. Statements that mention empowerment and agency were agreed upon with the highest agreement as relevant recovery indicators.

**Table 3.** Indicators that a person is progressing in their recovery: statements with more than 80% of agreement

Statement^a^	Agreement (%)	Round
27. The person is empowered (i.e., taking control of their own life and making autonomous decisions).	97.3	2
28. The person exerts agency in their own life (i.e., independent capability or ability to act on one’s will).	94.6	2
29. The person engages in activities that he/she enjoys.	91.9	2
30. The person has or is improving self-awareness (e.g., strengths and weaknesses, needs and preferences).	89.9	3
31. The person manages personal suffering and stress.	89.9	3
32. The person makes sense of his/her own experience (e.g., self-acceptance).	89.2	2
33. The person has a supportive social network (e.g., family and friends).	89.2	2
34. The person feels more satisfaction with life and general accomplishment.	87.8	2
35. The person feels there is meaning in their life.	87.8	2
36. The person resumes activities or has an interest in new ones.	87.8	2
37. The person acts with self-determination about their goals, needs, activities, treatment, etc.	87.8	2
38. The person overcomes the adverse effects of psychiatric drugs and treatments.	87.0	3
39. The person constructs their personal and social identity beyond the mental health problem (e.g., leaving the sick role).	86.5	2
40. The person has hope for recovery, the future, or achieving personal goals.	86.5	2
41. The person is able to enjoy their own life (e.g., enjoying hobbies, being happy in society and having a sense of humour).	86.5	2
42. The person learns to live and enjoy themselves despite their diagnosis/symptoms.	86.5	2
43. The person is aware of their legal capacity (i.e., the right to have rights and to be able to exercise them).	85.1	2
44. The person feels accepted and valued by others.	85.1	2
45. The person has autonomy and independence (e.g., depending less on professionals and family members).	85.1	2
46. The person copes with critical aspects of the recovery path (e.g., conflicts, symptoms, difficulties).	84.1	3
47. The person is assertive (e.g., establishing healthy boundaries with people and asserting their own opinion).	84.1	3
48. The person feels confident about their capabilities.	83.8	2
49. The person becomes an active member of the community (e.g., participation, a sense of belonging in the community).	82.6	3
50. The person knows helpful recovery strategies.	82.4	2
51. The person achieves goals or is progressing towards them.	81.2	3
52. The person has a healthy lifestyle (e.g., eating well, exercising, good sleep hygiene, does not abuse substances).	81.2	3
53. The person feels a connection with others.	81.2	3
54. The person maintains a meaningful life according to their major goals.	81.1	2
55. The person has a positive self-concept.	81.1	2
56. The person is improving interpersonal relationships.	81.1	2
57. The person has their basic financial needs covered.	81.1	2

a Statements rated as ‘relevant’ or ‘very relevant’ by more than 80% of the participants after the second and third rounds.

Table 4 shows the eight statements agreed upon by at least 80% of participants as being ‘relevant’ or ‘very relevant’ factors that facilitate recovery (i.e., Question 3). The table includes the percentage of agreement regarding the relevance of each statement and the round number in which consensus was reached. The statement with the highest agreement percentage highlights the importance of a supportive and respectful social environment to facilitate recovery.

**Table 4.** Factors that facilitate recovery: statements with more than 80% of agreement

Statement^a^	Agreement (%)	Round
58. Having a supportive and respectful social environment (e.g., emotionally stable, safe, non-paternalistic).	98.6	3
59. Having support from significant others (e.g., family, friends).	94.2	3
60. Having ethical health professionals (e.g., free of stigma, free of conflict of interests, aware of their limitations).	92.8	3
61. Living in an inclusive and equitable society (e.g., with space for diversity).	91.3	3
62. Overcoming stigma and self-stigma (e.g., the sick role).	89.9	3
63. Having a holistic approach to treatment, not only focused on symptoms.	87.0	3
64. Having financial security (e.g., housing, food).	84.9	2
65. Having a guarantee of human rights (e.g., access to justice).	83.6	2

a Statements rated as ‘relevant’ or ‘very relevant’ by more than 80% of the participants after the second and third rounds.

Finally, 12 statements were agreed upon by at least 80% of participants as ‘relevant’ or ‘very relevant’ factors that hinder recovery (i.e., Question 4). Table 5 presents the agreement percentage and the round number of consensuses. Findings with the highest agreement refer to coercion, discrimination and the lack of support from significant others.

**Table 5.** Factors that hinder recovery: statements with more than 80% of agreement

Statement^a^	Agreement (%)	Round
66. Being coerced (e.g., interfering with one’s own decisions).	95.7	3
67. Suffering from gender or any other discrimination (e.g., racism, ageism, homophobia and transphobia, classicism, ableism).	95.7	3
68. Lack of support from significant others (e.g., family, friends).	95.7	3
69. Lack of self-determination opportunities (i.e., lack of opportunities for taking positive risks).	94.2	3
70. Feeling that life is meaningless.	91.3	3
71. Suffering from social exclusion.	90.4	2
72. Suffering from social stigma (e.g., from professionals, family, friends).	89.0	2
73. Receiving psychiatric violence (e.g., forced drugging, electroshock, incarceration, restraint, solitary confinement).	86.3	2
74. Rights deprivation (e.g., Convention on the Rights of Persons with Disabilities – CRPD – violations).	84.9	2
75. Having financial insecurity (e.g., limited access to housing or food).	82.2	2
76. Having adverse environmental factors (e.g., war, geographical isolation).	80.8	2
77. Having a negative social environment (e.g., stressful, disempowering, blaming, miscommunicating).	80.8	2

a Statements rated as ‘relevant’ or ‘very relevant’ by more than 80% of the participants after the second and third rounds.

## Discussion

In this study, we aimed to achieve a consensus on the key aspects of recovery in mental health from the perspective of movements of users and survivors of psychiatry at an international level. We identified statements defining recovery, indicators that a person is progressing in their recovery and factors facilitating or hindering recovery. Our findings align with previous literature and contribute to understanding recovery, with implications for service implementation, policy development and guaranteeing respect for users’ rights.

### The definition of recovery

Consensus statements identifying what recovery in mental health means (i.e., Question 1) highlight both idiosyncratic and social aspects. Our findings show that recovery in mental health is a personal path that includes empowerment, safety, rights, good feelings about oneself, well-being, self-awareness, support, growth, hope, coping, overcoming self-stigma, rebuilding life, moving away from the biomedical model and having a fulfilling life.

The CHIME framework (Leamy *et al.*, 2011) is fully represented in the consensus statements and aligns with its frequent use in describing personal recovery, as shown in previous studies (Kuek *et al.*, 2020). In contexts beyond Western societies, the CHIME framework has served as a reference in Asian cultures (Murwasuminar *et al.*, 2023), highlighting self-esteem (Chang and Chen, 2022), agency (Suryani *et al.*, 2022), support (Kuek *et al.*, 2022) and the ability to live with the mental health problems (Kuek *et al.*, 2024). Similarly, agency has been also described within African cultures (Kpanake, 2018), while advocating for rights has been emphasized in Latin America (Ardila-Gómez *et al.*, 2019). Despite the widely documented cultural differences in the definition of recovery, our results show that achieving an international consensus on the key aspects defining recovery is attainable.

The statements defining recovery agreed upon in this study also are similar to those from the study of Law and Morrison (2014), which emphasized quality of life and feeling good. Nevertheless, they reported information related to a biomedical approach (i.e., symptoms) in the same direction as other studies (Gopal *et al.*, 2019; Piat *et al.*, 2009), which differs from our Delphi study that did not define recovery based on symptoms, nor did they refer to a pre-illness functioning. This may be due to differences in participants’ access to alternative paradigms. While Law and Morrison’s study recruited participants through mental health services, our participants were mainly recruited from users’ and survivors’ organizations. Therefore, they may be more likely to have assimilated insights from the recovery paradigm compared to those who are not involved in these movements (Sampietro *et al.*, 2022).

The findings in our study underline new aspects related to the role of human rights and the living conditions of people with psychosocial disabilities, which have been incorporated more recently into the SPICE model of recovery (Vera San Juan *et al.*, 2021). Notably, statements that point to wellness and joy are related to literature that highlights the intersection between the recovery approach and well-being research by leaving behind the traditional biomedical discourse and focusing on living well (Slade *et al.*, 2017).

Our results shed new light on users’ and survivors’ perspectives about recovery, which involves moving away from the biomedical perspective and supporting collective rights. The agreement reached in this study indicates the possibility of recovery through a new paradigm away from the dominant biomedical model and embracing a human-rights approach following current recommendations of the United Nations Human Rights Council (2020). In this regard, although the CHIME framework is effective for defining recovery from the perspective of users and survivors, this study highlights the need to introduce a new process, which could be called ‘Transition’ to signify moving away from the biomedical model approach and advocating for collective rights. Considering the essential role of the experiences of users and survivors in understanding recovery in mental health (Kidd *et al.*, 2014), it is recommended to integrate the ‘Transition’ process into future research.

In summary, users and survivors mainly agree that recovery involves empowerment and self-determination to create a fulfilling life in which they feel safe and their rights are respected; therefore, services and policies should implement actions addressing these aspects.

### The indicators that a person is progressing in their recovery

Consensus statements indicating that a person is progressing in their recovery (i.e., Question 2) include empowerment, active participation, self-awareness, coping, assertiveness, self-confidence, well-being, positive self-concept, feelings of joy, satisfaction, a fulfilling life, goal achievement, learning how to live, meaning in life, constructing identity, hope, overcoming effects of psychiatric drug and treatments, having financial needs covered, having a connection with others and being supported.

The consensus statements for defining recovery align with previous literature and introduce new aspects for consideration. Some of the statements are related to the CHIME processes (Leamy *et al.*, 2011), with the most agreed-upon statement highlighting empowerment. This underscores the need to challenge power dynamics and opposes involuntary treatments or actions that strip users of legal capacity, self-determination and autonomy (United Nations Human Rights Council, 2020).

In contrast to Law and Morrison (2014), our study did not achieve consensus on aspects related to symptoms but identified new recovery indicators. First, users and survivors need to be aware of their legal capacity, reinforcing the need for rights-based policies and training for health professionals. Second, the study highlights the need to recover from the effects of psychiatric treatment. Previous reports have pointed to the negative consequences of medicalization (United Nations Human Rights Council, 2020). Services need to guarantee that users and professionals are aware of the effects of psychiatric drugs during treatment. It is essential to consider and truly believe what users say about their experience with psychiatric drugs or treatments and to provide them with options that include support to discontinue medication if desired (United Nations Human Rights Council, 2020). Third, covering basic financial needs is essential, highlighting the prior role of policies to create social laws that support people with low economic resources. Recent studies have also analysed legal, political and economic factors of recovery as relevant considerations from the perspective of users (Vera San Juan *et al.*, 2021). In our study, participants agreed that having financial needs covered is an indicator of recovery, emphasizing the importance of addressing this issue in political agendas and services for a better understanding of mental health. In summary, the statements agreed upon as indicators of recovery from the perspective of users and survivors of psychiatry call for shared responsibility among services, policies and society.

### The factors that facilitate and hinder recovery

Facilitators and hindrances seem to be extremes of the same scale, for example, ‘having financial security’ vs. ‘having financial insecurity’. The statements that achieved consensus to describe what facilitates recovery (i.e., Question 3) pertain to social, political and care-related aspects. On the other hand, the statements that achieved consensus on what hinders recovery (i.e., Question 4) highlight personal factors alongside social, political and care-related ones. Most of these facilitators and hindrances underscore external factors (i.e., outside the control of users and survivors) that need to be addressed in political strategies, specific community programmes and person-centred and recovery-oriented services. Despite services being in the process of change, it seems that coercion is still a frequent problem reported that tends to be unaware by mental health professionals (Perry *et al.*, 2017; Stasiulis *et al.*, 2021).

Our results highlight the relevance of social factors in mental health recovery in the same direction as previous findings (Kuek *et al.*, 2022; Tsoi *et al.*, 2021; Xu *et al.*, 2019). Moreover, several of our statements also reflect broader issues prevalent in individuals with mental health problems in many other cultures, such as stigma in Black and minority ethnic communities in Western countries (Leamy *et al.*, 2011), discrimination in Asian cultures (Kuek *et al.*, 2020), financial challenges in Latin America (Ardila-Gómez *et al.*, 2019) and concerns for human rights in Africa (Kleintjes *et al.*, 2013). While spirituality is valued in many cultures such as in Asian, African and Latin American cultures (Caplan, 2019; Kpanake, 2018; Suryani *et al.*, 2022), it did not reach consensus (see the online Supplementary Table S3). This may stem from both the specific characteristics of the study’s sample and the potential for religious traditions to perpetuate stigma (Caplan, 2019; Kuek *et al.*, 2020).

In summary, our results shed light on the demand from users and survivors for an ethical and holistic approach without stigma, coercion or violence. Given that the biomedical approach has been criticized for potentially masking these issues (United Nations Human Rights Council, 2020), it is imperative, from the viewpoint of users and survivors, for the healthcare community to cease rationalizing certain protocols solely based on symptoms. Instead, the focus needs to shift towards embracing a range of perspectives to understand human distress and psychosocial diversity.

## Strengths and limitations

Incorporating the lived experiences of users and survivors from international movements is the main strength of this study. The first round of open-ended questions enabled us to directly gather insights from participants’ lived experiences, supplementing the information typically obtained solely from published reports. Notably, all contributions, including those from a minority of participants, were included in the list of statements for the second round and thus subjected to the consensus process.

Achieving participation from five different continents and obtaining a diverse sample was made possible by combining various recruitment strategies. Although we made considerable efforts to encourage and diversify participation, less than a third of users and survivors invited from the initial recruitment list participated in the study. Moreover, a large proportion of participants were from Southern Europe, specifically from Spain. This predominance can be attributed to the fact that nine out of the organizations that agreed to distribute the study invitation were based in Spain. Nonetheless, including participants from this region broadens the perspective of studies which until now have mainly been conducted in English-speaking countries (Slade *et al.*, 2012). Additionally, the strategy of using an online questionnaire in the Delphi process might have been an obstacle to obtaining more participation due to limited access to the Internet and online services in some countries.

Despite the limitations, our study could guide users and survivors in defining their recovery journey (Kuek *et al.*, 2024), as well as enhance the understanding of professionals and families about how to better support users in recovery. This involves recognizing recovery indicators and acknowledging both facilitators and hindrances in the recovery journey.

Considering recent studies suggesting that different mental disorders may influence personal recovery in diverse ways (Jagfeld *et al.*, 2021; Luciano *et al.*, 2022; Richardson and Barkham, 2020), further investigation is warranted to explore the impact of diagnostic categories on mental health recovery.

## Conclusion

This study represents the first consensus on the key aspects of recovery in mental health from the perspective of movements of users and survivors of psychiatry. Results align with frameworks like CHIME, highlighting universal recovery processes. The ‘Transition’ process was emphasized, advocating for a rights-based approach. Recovery indicators included recovering from the effects of psychiatric treatment, being aware of one’s legal capacity and having financial security, enriching the understanding of recovery beyond traditional biomedical perspectives. Factors facilitating and hindering recovery underscore holistic approaches to mental health and address external factors such as stigma and socioeconomic disparities. The findings inform service delivery, policy and advocacy, enhancing global mental healthcare. They lay the groundwork for future research tailored to diverse contexts, exploring innovative perspectives on diagnosis, intervention and evaluation.

## Supporting information

Guerrero et al. supplementary material Guerrero et al. supplementary material

## Data Availability

The participants in this study were not asked for consent to share their data publicly; therefore, the supporting data are not available.

## Supplementary material

The supplementary material for this article can be found at https://doi.org/10.1017/S2045796024000490.
