# Supplementary material for: Let’s talk about recovery in mental health: an international Delphi study of experts by experience
Source: Epidemiol Psychiatr Sci. 2024 Sep 24;33:e41. doi: 10.1017/S2045796024000490 (PMC11464929; doi:10.1017/S2045796024000490)

**SUPPLEMENTARY MATERIAL**

- Table S1. User and survivor recruitment strategies.
- Table S2. Examples of conversion from category to statement.
- Table S3. Statements discarded.
- Figure S1. Example of feedback presented to participants in the third round.

**Table S1.** User and survivor recruitment strategies.

| **No.** | **Sampling** | **Period** | **Target** | **Description** |
| --- | --- | --- | --- | --- |
| #1 | Convenience | June-July 2022 | Organizations of users and survivors and their members | Step 1. A peer researcher from a local organization of experts by experience compiled a list of users and psychiatric survivors’ organizations.  Step 2. Board members of the organizations were identified.  Step 3 A list of members of the organizations was compiled.  Step 4. Users identified in steps 2 and 3 were contacted to informally propose additional users and survivors within their network.  Step 5. Users and survivors who have collaborated in previous studies with the research team were identified.  Results: A contact list of 53 organizations and 99 users and survivors from 30 countries across five continents around the world was established. |
| #2 | Convenience | July 2022 | Users and survivors: authors of technical or scientific publications from a situated position of an expert by lived experience | Step 1. An electronic search in Google Scholar was conducted using the search terms ‘users’ and ‘survivors’.  Step 2. The first 100 publications were selected, and authors were identified.  Step 3. For all the authors, the publication itself was screened to determine if they were speaking from a situated position as an expert by lived experience. If this was not clear from the text, the author was searched on the internet to find out about their activity and/or other publications in which their role was made clear.  Results: A contact list of 17 users and survivors who had not been identified in search strategy #1. |
| #3 | Convenience | July 2022 | Users and survivors: contributors to WHO QualityRights materials | Step 1. WHO QualityRights initiative materials published in 2019 were searched and downloaded [https://www.who.int/publications/i/item/who-qualityrights-guidance-and-training-tools].  Step 2. Contributors were identified.  Step 3. Each contributor's name was searched on the internet to find out who identified themselves as an expert by lived experience.  Results: 12 participants who had not been identified in search strategies #1 and #2. |
| #4 | Snowball | September 2022 | Users and survivors from the initial contact list | Step 1. Invitations were sent to 53 organizations and 128 users and survivors. Organizations were required to share information about the study with their affiliates and to advertise the study to other organizations worldwide.  Step 2. Users and survivors who consented to participate (*n* = 34) were asked to share the contact information of other users and survivors who might be interested in participating.  Results: Not known, since snowball sampling was used. |

**Table S2.** Examples of conversion from category to statement.

| **Questions** | **Category** | **Statement** | **Consideration** |
| --- | --- | --- | --- |
| Question 1 | No psychiatric drugs or minimal intake. | Being free of psychiatric drugs or consuming just a minimal intake. | Statement discarded (see Table S3) |
| Question 2 | Working on resilience. | The person is becoming more resilient. | Statement discarded (see Table S3) |
| Question 3 | Activism/Associationism. | Being an activist or part of an association. | Statement discarded (see Table S3) |
| Question 4 | Misdiagnosis. | Receiving a wrong mental health diagnosis. | Statement discarded (see Table S3) |

**Table S3.** Statements discarded.

| **Question** | **Statement ^a^** | **Agreement %** | **Round** |
| --- | --- | --- | --- |
| Question 1: Definition of recovery | Having adequate social skills (e.g., being empathic with others). | 72.5 | 3 |
|  | Practising self-care (e.g., brushing teeth, leisure). | 68.8 | 2 |
|  | Developing assertiveness. | 68.8 | 2 |
|  | Since recovery is an idiosyncratic experience, the meaning is subjective and defined by oneself. | 67.5 | 2 |
|  | Having rules and routines that make life easier. | 66.2 | 2 |
|  | Having adequate occupational functioning (e.g., being employed, studying). | 64.9 | 2 |
|  | Living according to one’s community. | 62.3 | 2 |
|  | Having adequate cognitive functioning (e.g., ability to memorize, to learn). | 61.0 | 2 |
|  | Being free of psychiatric drugs or consuming just a minimal intake. | 53.3 | 2 |
|  | Having support from mental health professionals. | 50.7 | 2 |
|  | Showing clinical improvement (i.e., overcoming symptoms). | 48.1 | 2 |
|  | Accepting the illness and its limitations. | 48.1 | 2 |
|  | It is a term that must no longer be used because it blames people and ignores human rights violations. | 37.7 | 2 |
| Question 2: Indicators that a person is progressing in their recovery | The person overcomes stigma and discrimination. | 79.7 | 3 |
|  | The person is becoming more resilient. | 78.3 | 3 |
|  | The person is able to ask for help. | 78.3 | 3 |
|  | The person has less suffering (e.g., less agitation or discomfort). | 76.8 | 3 |
|  | The person feels satisfied with their social life. | 73.9 | 3 |
|  | The person is open to learning new ways of looking at the world. | 68.9 | 2 |
|  | The person expresses that recovery is taking place. | 67.6 | 2 |
|  | The person has commitment capacity (i.e., fulfilling tasks and commitments). | 66.2 | 2 |
|  | The person trusts others and considers their points of view and experiences. | 66.2 | 2 |
|  | The person has less reliance on the biomedical model (e.g., depending less on psychiatric drugs) | 63.5 | 2 |
|  | The person forgives himself/herself and others. | 63.5 | 2 |
|  | The person has an active occupation (e.g., employment or study) or is on course to achieving this. | 58.1 | 2 |
|  | The person is reducing their use of mental health services. | 52.7 | 2 |
|  | The person uses suitable non-verbal communication (e.g., tone of voice, facial expression, smile). | 50.0 | 2 |
|  | The person is psychologically stable. | 50.0 | 2 |
|  | The person has fewer symptoms. | 47.3 | 2 |
|  | Since recovery is a personal process, general indicators of recovery cannot be identified. | 32.4 | 2 |
|  | The person is living according to social norms. | 32.4 | 2 |

| Question 3: Factors that facilitate recovery | Being an activist or part of an association. | 69.9 | 2 |
| --- | --- | --- | --- |
|  | Having personal autonomy (i.e., independent living). | 68.5 | 2 |
|  | Having self-determination (i.e., making decisions about one's identity and future without others' control, taking positive risks). | 68.5 | 2 |
|  | Receiving hope from providers and the community. | 68.5 | 2 |
|  | Being aware of the validity of one´s own emotions and thoughts. | 65.8 | 2 |
|  | Receiving government support (i.e., having basic needs covered, suppression of violence and coercion). | 65.8 | 2 |
|  | Receiving peer support. | 64.4 | 2 |
|  | Making sense of one´s own experience (e.g., self-acceptance). | 63.0 | 2 |
|  | Receiving information about one´s rights (e.g., advance directives, recovery plan). | 63.0 | 2 |
|  | Having meaningful and realistic goals (i.e., having a purpose in life). | 61.6 | 2 |
|  | Having an inclusive occupation (e.g., job, study). | 61.6 | 2 |
|  | Receiving trauma-informed care. | 61.6 | 2 |
|  | Enjoying safe intimate relationships. | 60.3 | 2 |
|  | Having time for oneself (e.g., for awareness, creating meaning, recovering). | 58.9 | 2 |
|  | Participating in meaningful and enjoyable activities (e.g., social activities, professional activities). | 58.9 | 2 |
|  | Receiving adequate therapeutic interventions (i.e., based on safe and supportive relationships, a solid treatment alliance, and avoiding a strong medical reductionistic model). | 58.9 | 2 |
|  | Having close or social referents of recovery. | 58.9 | 2 |
|  | Practising self-care (e.g., brushing teeth, leisure). | 57.5 | 2 |
|  | Living positive experiences. | 57.5 | 2 |
|  | Participating in a mutual aid group. | 57.5 | 2 |
|  | Having proper, regular, affordable, and easily accessible care services. | 57.5 | 2 |
|  | Receiving adequate and monitored medication (e.g., not overmedicalization). | 57.5 | 2 |
|  | Having self-confidence. | 56.2 | 2 |
|  | Having trained health providers. | 56.2 | 2 |
|  | Having self-awareness (e.g., strengths and weaknesses, needs and preferences, progress and achievements). | 53.4 | 2 |
|  | Noticing possible trigger factors. | 53.4 | 2 |
|  | Creating advance directives. | 53.4 | 2 |
|  | Having beneficial personality factors (e.g., emotional stability, adaptiveness). | 52.1 | 2 |
|  | Giving and receiving support when interacting with others. | 52.1 | 2 |
|  | Developing abilities and skills (e.g., social skills). | 50.7 | 2 |
|  | Having spirituality. | 50.7 | 2 |
|  | Being accepted and valued by others. | 50.7 | 2 |
|  | Being an active member of the community (e.g., a sense of belonging and citizenship). | 50.7 | 2 |
|  | Being collaborative with the treatment. | 50.7 | 2 |
|  | Receiving the right mental health diagnosis. | 50.7 | 2 |
|  | Having a healthy lifestyle (e.g., healthy diet, physical activity). | 49.3 | 2 |
|  | Accepting treatment options (e.g., psychosocial interventions, psychotherapy, psychoeducation). | 49.3 | 2 |
|  | Having good physical health. | 48.0 | 2 |
|  | Having medication compliance. | 48.0 | 2 |
|  | Having fewer symptoms. | 46.6 | 2 |
|  | Since recovery is a personal process, general facilitators of recovery cannot be identified. | 45.2 | 2 |
|  | Having routines (e.g., structured days). | 42.5 | 2 |
|  | Having pet(s). | 41.1 | 2 |
| Question 4: Factors that hinder recovery | Lack of agency (e.g., not having opportunities to make autonomous decisions). | 69.9 | 2 |
|  | Lack of information about one´s rights (e.g., human rights, advance directives, recovery plan). | 68.5 | 2 |
|  | Lack of personal autonomy (i.e., not having independent living). | 67.1 | 2 |
|  | Lack of communication with others (e.g., feeling silenced, when one’s own experience is not valued). | 67.1 | 2 |
|  | Lack of trained health providers. | 67.1 | 2 |
|  | Lack of professional support for demedicalization. | 65.8 | 2 |
|  | Lack of peer support from the services. | 65.8 | 2 |
|  | Having self-stigma (e.g., the sick role). | 64.4 | 2 |
|  | Lack of opportunities for empowerment. | 64.4 | 2 |
|  | Having a perspective of chronicity. | 64.4 | 2 |
|  | Lack of adequate and monitored medication (e.g., overmedicalization). | 64.4 | 2 |
|  | Lack of care in our social environment (i.e., institutionalization). | 64.4 | 2 |
|  | Using diagnostic categories (e.g., DSM, ICD) or any tool from the biomedical model. | 64.4 | 2 |
|  | Having low psychological resources (e.g., low hope, self-esteem, self-efficacy, communication skills, coping strategies). | 63.0 | 2 |
|  | Having adverse effects of psychiatric drugs. | 63.0 | 2 |
|  | Having persistent unusual experiences/psychosis. | 63.0 | 2 |
|  | Lack of a psychosocial disability perspective from society. | 63.0 | 2 |
|  | Having persistent and acute symptoms. | 61.6 | 2 |
|  | Lack of proper, regular, affordable, and easily accessible care services. | 60.3 | 2 |
|  | Receiving a wrong mental health diagnosis. | 60.3 | 2 |
|  | Having limited opportunities for engaging in meaningful activities. | 60.3 | 2 |
|  | Consuming substances (e.g., alcohol, tobacco, marijuana, cocaine). | 58.9 | 2 |
|  | Lack of a healthy lifestyle (e.g., insufficient sleep, sedentarism). | 58.9 | 2 |
|  | Lack of government support (e.g., lack of investment in prevention and investigation). | 58.9 | 2 |
|  | One’s own mental health history (e.g., trauma experiences). | 57.5 | 2 |
|  | Lack of self-care awareness. | 53.4 | 2 |
|  | Lack of medication compliance. | 50.7 | 2 |
|  | Having limited occupational opportunities (e.g., job, study). | 50.7 | 2 |
|  | Lack of self-awareness of one´s own mental health experience. | 48.0 | 2 |
|  | Lack of cooperation with caretakers. | 41.1 | 2 |
|  | Having unrealistic goals. | 38.4 | 2 |

^a^ Statements discarded after being rated as ‘relevant’ or ‘very relevant’ by less than 70% of the participants in the second round or by less than 80% of the participants in the third round.

**Figure S1.** Example of feedback presented to participants in the third round.


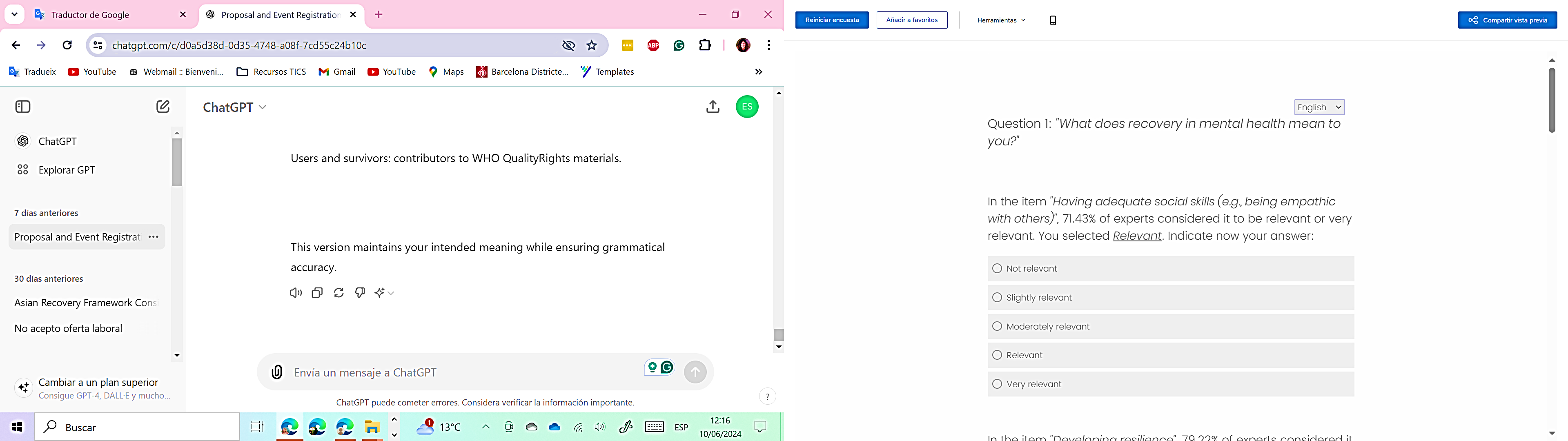

Supplement: Guerrero et al. supplementary material [file S2045796024000490sup001.docx]
